# Developing genome-reduced *Pseudomonas chlororaphis* strains for the production of secondary metabolites

**DOI:** 10.1186/s12864-017-4127-2

**Published:** 2017-09-11

**Authors:** Xuemei Shen, Zheng Wang, Xianqing Huang, Hongbo Hu, Wei Wang, Xuehong Zhang

**Affiliations:** 1 State Key Laboratory of Microbial Metabolism, School of Life Sciences and Biotechnology, Shanghai Jiao Tong University, No. 800 Dongchuan Road, Shanghai, 200240 People’s Republic of China; 2 Beijing Key Laboratory of Nutrition, Health and Food Safety, Nutrition and Health Research Institute, COFCO Corporation, No.4 Road, Future Science and Technology Park South, Beijing, 102209 People’s Republic of China

**Keywords:** *Pseudomonas chlororaphis*, Secondary metabolite, Comparative genomics, Markerless deletion, Reduced-genome

## Abstract

**Background:**

The current chassis organisms or various types of cell factories have considerable advantages and disadvantages. Therefore, it is necessary to develop various chassis for an efficient production of different bioproducts from renewable resources. In this context, synthetic biology offers unique potentialities to produce value-added products of interests. Microbial genome reduction and modification are important strategies for constructing cellular chassis and cell factories. Many genome-reduced strains from *Escherichia coli*, *Bacillus subtilis*, *Corynebacterium glutamicum* and *Streptomyces,* have been widely used for the production of amino acids, organic acids, and some enzymes. Some *Pseudomonas* strains could serve as good candidates for ideal chassis cells since they grow fast and can produce many valuable metabolites with low nutritional requirements and strong environmental adaptability. *Pseudomonas chlororaphis* GP72 is a non-pathogenic plant growth-promoting rhizobacterium that possesses capacities of tolerating various environmental stresses and synthesizing many kinds of bioactive compounds with high yield. These include phenazine-1-carboxylic acid (PCA) and 2-hydroxyphenazine (2-OH-PHZ), which exhibit strong bacteriostatic and antifungal activity toward some microbial pathogens.

**Results:**

We depleted 685 kb (10.3% of the genomic sequence) from the chromosome of *P. chlororaphis* GP72(*rpeA*-) by a markerless deletion method, which included five secondary metabolic gene clusters and 17 strain-specific regions (525 non-essential genes). Then we characterized the 22 multiple-deletion series (MDS) strains. Growth characteristics, production of phenazines and morphologies were changed greatly in mutants with large-fragment deletions. Some of the genome-reduced *P. chlororaphis* mutants exhibited more productivity than the parental strain GP72(*rpeA*-). For example, strain MDS22 had 4.4 times higher production of 2-OH-PHZ (99.1 mg/L) than strain GP72(*rpeA*-), and the specific 2-OH-PHZ production rate (mmol/g/h) increased 11.5-fold. Also and MDS10 had the highest phenazine production (852.0 mg/L) among all the studied strains with a relatively high specific total phenazine production rate (0.0056 g/g/h).

**Conclusions:**

In conclusion, *P. chlororaphis* strains with reduced genome performed better in production of secondary metabolites than the parent strain. The newly developed mutants can be used for the further genetic manipulation to construct chassis cells with the less complex metabolic network, better regulation and more efficient productivity for diverse biotechnological applications.

**Electronic supplementary material:**

The online version of this article (10.1186/s12864-017-4127-2) contains supplementary material, which is available to authorized users.

## Background

The exploration of minimal bacterial gene set has become a research hotspot since the smallest known bacterial genome of any free-living organism was fully sequenced, which is about 470 protein-coding genes in a 580 kb *Mycoplasma genitalium* genome [1]. Until now, great progresses have been made in certain model strains under particular growth conditions, e.g., 303 essential genes in *Escherichia coli* [2], 261 (including 2 functional RNAs genes) in *Bacillus subtilis* [3], 335 in *P. aeruginosa* species [4], and 14.7% of the *Streptococcus agalactiae* genes are essential and critical [5]. Herein, the Database of Essential Genes (DEG) provides comprehensive information on essential genes of each strain [6], which could guide further research on other strains or species. Essential genes play important roles in understanding the last universal common ancestor [7], determining minimal gene set for cellular life [8], identifying therapeutic candidates [9], and designing chassis cells from the perspective of synthetic biology [10].

The synthetic biology principle facilitates the construction of microbial cell factories or chassis cells for biotechnological applications [11]. An ideal bacterial chassis used in biotechnology should have enough genetic information to maintain the basic biological functions, such as self-maintenance, robust cell growth and strong stress resistance, and simplified genome to make effective use of the intracellular energy [12]. The microbial genome reduction and modification are important strategies for constructing cellular chassis, such as *E. coli* strains ∆16 [13] and MGF-01 [14]**,** *B. subtilis* strain MG1M [15], *C. glutamicum* strains [16], *S. avermitilis* SUKA17 [17] and *S. coelicolor* M145 [18]. Great achievements have been made in these model organisms due to their well-characterized background or easily genetic manipulation [19].

New chassis cells from different microbial genotypes should also be needed to produce complex molecules and valuable secondary metabolites, and sometimes they have to tolerate substantial environmental stresses [20]. A recent study shed light that several species of the genus *Pseudomonas* could serve as good candidates for chassis cells [21]. *Pseudomonads* are endowed with extensive metabolic and physiological diversity and can tolerate various stresses, which make them ideal resources of bacterial chassis [21]. *P. putida* with 4.3% eliminated genome was proved to be an enhanced host to express heterologous genes (GFP-LuxCDABE reporter system) [22]. This largely owns to *Pseudomonas* remarkable biodegradation capabilities accompanied by high tolerance to organic solvents. However, a biological chassis from *Pseudomonas* for the production of secondary metabolites has not been reported yet.

*P. chlororaphis* strains without virulence factors are well known plant growth-promoting rhizobacteria and widely used in agriculture and production of bio-pesticides [23]. *P. chlororaphis* GP72 was isolated from green pepper rhizosphere [24]. Previous studies indicated that this strain could withstand both endogenous and exogenous stresses, and synthesize different type of phenazines, including PCA, 2-hydroxy-phenazine-1-carboxylic acid (2-OH-PCA) and 2-OH-PHZ [25, 26]. Phenazines are a group of over 100 natural and more than 6000 synthetic nitrogen-containing heterocyclic compounds [27]. Strains of *Pseudomonas* and *Streptomyces* are main phenazine producers, and formers are used to produce simple phenazine compounds, while latters tend to generate more diverse and complex molecules [28]. Phenazine metabolites make enormous contributions to agriculture due to their broad spectrum antibiotic properties [29], in industrial application owing to the electron transfer ability [30], and in health area because of the anti-cancer activity [31]. In *P. chlororaphis* GP72, the production of 2-OH-PHZ has been greatly improved through genetic engineering [32–34], indicating that strain GP72 has noteworthy potential for enhanced production of secondary metabolites. Herein, we aimed to implement a comparative genomic approach together with genome mining strategy, to design and construct valuable genome-reduced *Pseudomonas* mutants.

## Results

### Computational prediction of deletion targets and set of essential genes

Maximal deletion regions on the chromosome of *P. chlororaphis* were selected using comparative genomics between *P. chlororaphis* GP72 and *P. stutzeri* A1501. As *P. stutzeri* strains are nitrogen-fixing root-associated bacteria harboring relative small genomes among pseudomonads [35], and strain A1501, a model organism of this species [36], is phylogenetically distant from strain GP72 [25]. Conserved genes between them are likely to be essential [37], while strain-specific ones are nonessential. Previous studies indicated that strain GP72 has six secondary metabolite gene clusters which cannot be found in the genome of strain *P. stutzeri* A1501 [25, 26]. So the five clusters, except the phenazine operon which can be used for testing the production capacity of secondary metabolites in mutants, can be eliminated firstly. Based on the in silico subtractive hybridization analysis via mGenomeSubtractor online software [38], there were 714 conserved genes and 3351 strain-specific ones in the genome of strain GP72. Therefore, regions with strain-specific genes longer than 15 kb were selected as candidate deletion regions, which provide a framework for the design of deletion targets.

Chassis cells must be efficient and profitable, which contain essential genes to sustain bacterial vitality, and set of favorable and selected genes for industrial use [39]. So those selected candidate regions were checked for the presence of predicted essential genes. The predicted essential gene was selected when the corresponding protein was conserved at least in eight of 15 essential gene data sets using the basic local alignment search tool (BLAST) under the threshold of 35% identity [40]. These sets of essential genes were from 15 g-negative bacteria [6], including *P. aeruginosa* UCBPP-PA14, *E. coli* MG1655 II, *Burkholderia pseudomallei* K96243, *Acinetobacter baylyi* ADP1, *Shewanella oneidensis* MR-1, *Salmonella enterica* subsp. *enterica* serovar Typhimurium SL1344, *Haemophilus influenzae* Rd. KW20, *Francisella novicida* U112, *Caulobacter crescentus*, *Sphingomonas wittichii* RW1, *Vibrio cholerae* N16961, *Porphyromonas gingivalis* ATCC 33277, *Bacteroides fragilis* 638R, *Campylobacter jejuni* NCTC 11168 and *Helicobacter pylori* G27. Two hundred fifteen genes were predicted to be essential out of the total 6176 genes in the genome of strain GP72 (Additional file 1). In each essential gene set, the number of homologous genes that can be blasted in GP72 is shown in Additional file 2.

**Fig. 1:**
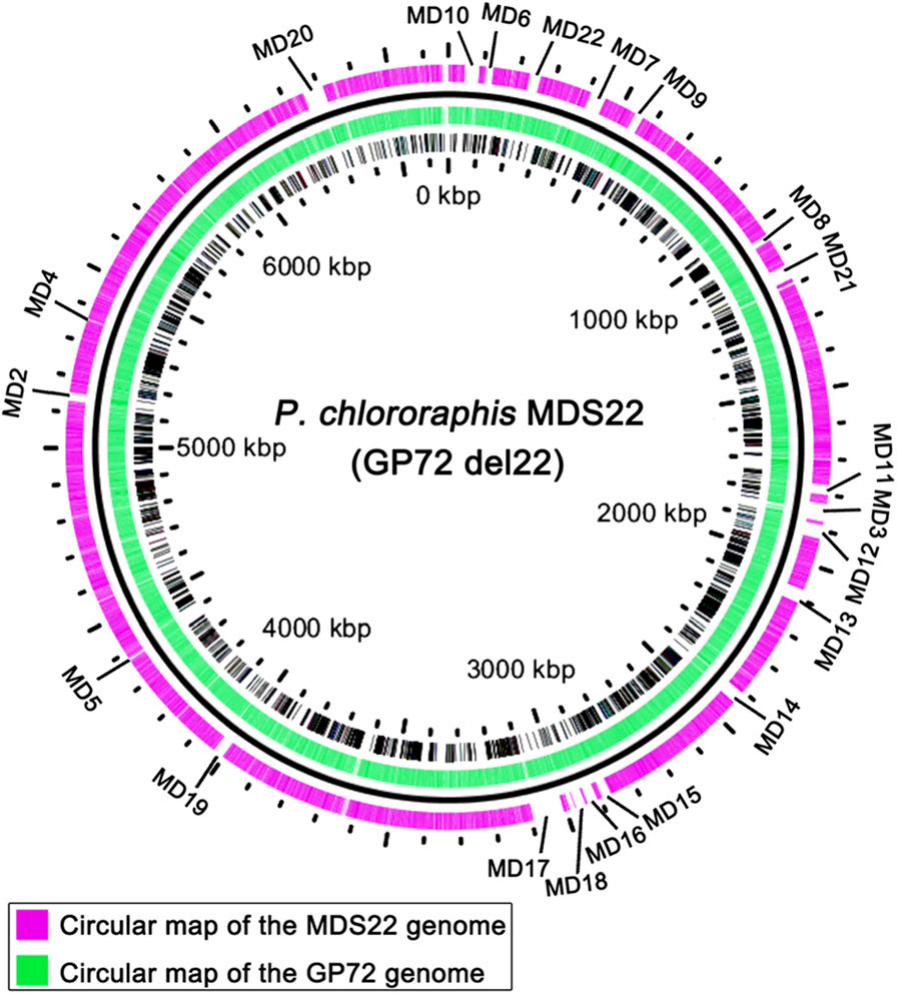
An overview of the reduced genome of *P. chlororaphis* MDS22. Deletions of MD1 through MD22 were illustrated on the circular map of the GP72 genome. The green and purple circles represent the genomes of GP72 and the largest deletion mutant (MDS22), respectively

Given the above alignments, 22 strain-specific genomic regions without essential genes were picked as deletion targets (Fig. 1). Based on the gene annotation, we hypothesized that the selected deletion regions could not affect the basic biochemistry and physiology quality of engineered strains.

### Construction of deletion strains

The selected regions were markerless deleted through homologous recombination via genetically modified plasmids, which were constructed using pK18 *mobsacB*. We sequentially deleted 22 multiple-deletion (MD) regions (MD1 to MD22) (Table 1), 575 genes in total (Additional file 3). And MDS22 lacked 685,750 base pairs (bp), accounting for 10.3% of the parent genome (6,663,241 bp). Deletion endpoint was designed in noncoding regions next to the deleted gene, or in some cases, it was in the deletion region, which may not affect the expression of remaining genes.

**Table 1:** List of large-scale chromosomal deletion mutants

Mutant	Multiple-deletion (bp)	Cumulative deletion		Genomic loci	Description
		(bp)	(%)		
MDS1	14,022	14,022	0.21	MOK_02716-MOK_02727	achromobactin operon
MDS2	21,614	35,636	0.53	MOK_04727-MOK_04735	*P.fluorescens* insecticidal toxin operon
MDS3	48,164	83,800	1.26	MOK_01688-MOK_01707	part of *pvd* operon
MDS4	2548	86,348	1.30	MOK_04926,MOK_04927	hydrogen cyanide operon
MDS5	6726	93,074	1.40	MOK_04030-MOK_04036	pyrrolnitrin operon
MDS6	19,167	112,241	1.68	MOK_00110-MOK_00130	includes *ccdA, azlC, tdcB, gss. mgtA*
MDS7	41,371	153,612	2.31	MOK_00373-MOK_00408	includes *ltaA, gbcAB, proXWV, betTIBA*
MDS8	18,221	171,833	2.58	MOK_00955-MOK_00974	includes *aurF, fabGZF, acpP, psyIR, ada*
MDS9	24,416	196,249	2.95	MOK_00497-MOK_00507	includes *pvdL, pvdS, atuR, atuA*
MDS10	38,000	234,249	3.52	MOK_00047-MOK_00091	includes *ligT, mmsB, araD, hyi, folA*
MDS11	26,771	261,020	3.92	MOK_01641-MOK_01663	includes *katG, vgrG, gstA, amiE, surE*
MDS12	37,633	298,653	4.48	MOK_01720-MOK_01754	includes *str, pea* operon, *aphA_2, mrdA_1*
MDS13	37,411	336,064	5.04	MOK_01897-MOK_01932	includes *matP, wbp* operon, *lpd3, cdsA_2*
MDS14	29,156	365,220	5.48	MOK_02207-MOK_02237	includes *hisPMQJ, dam*
MDS15	24,108	389,328	5.84	MOK_02626-MOK_02642	includes *dmpA, apr* operon, *pspAB, pueBA*
MDS16	28,273	417,601	6.27	MOK_02657-MOK_02685	includes *ssuBCA, aidB, uspA, puuC, dehII*
MDS17	87,173	504,774	7.58	MOK_02743-MOK_02813	includes *fabG, idnO, aofH, aidB, phoD, aofH*
MDS18	26,866	531,640	7.98	MOK_02690-MOK_02714	includes *gabT, narL, glnA_3, plcN*, FecR
MDS19	28,745	560,385	8.41	MOK_03695-MOK_03722	mostly related to hypothetical protein
MDS20	56,152	616,537	9.25	MOK_05764-MOK_05802	mostly related to hypothetical protein
MDS21	40,441	656,978	9.86	MOK_01057-MOK_01097	*czcABCRD* and genes with unknown function
MDS22	28,772	685,750	10.29	MOK_00230-MOK_00246	mostly encoding hypothetical protein

The deletion strategy we implemented was that genes responsible for biosynthesis of secondary metabolites were deleted as the first choice, including MD1–5. MDS1 was created by deleting the achromobactin operon synthesizing a temperature-regulated secondary siderophore [41]. Then the MDS2 was constructed based on MDS1 by removing MD2. Genes in MD2 are responsible for a *P. fluorescens* insecticidal toxin which exhibits oral insecticidal activity [42]. Another siderophore operon pyoverdine (Pvd, excluding *pvdL*, *pvdS* and *pvdY*) was deleted to design MDS3. MDS4 was constructed by deleting hydrogen cyanide gene cluster which cannot be found in the genome of A1501 but existed in many other pseudomonads [25]. The pyrrolnitrin operon [43] encoding the chlorinated phenylpyrrole compound based on L-tryptophan was also removed, resulting in the fifth mutant MDS5.

Afterwards, unnecessary metabolisms or transport genes of the strain when inoculated in the rich medium were selected for deletion (MD6–18). Those include genes related to amino acid metabolism or transport, carbohydrate metabolism or transport, inorganic ion transport, and signal transduction system. In MD6, four of the 21 genes may be related to threonine transport and metabolism based on the cluster of orthologous group (COG) prediction and nine of them have not been functionally predicted. The threonine dehydratase (TdcB) may play roles in threonine or serine degradation, but it is only expressed during anaerobic growth in the absence of glucose [44]. The osmoprotectant glycine betaine could be absorbed from the environment [45]. So its biosynthetic genes (MOK_00378–00406) in MD7 were removed. According to the gene annotation and metabolic pathway analysis, genes MOK_00955–00968 in MD8 might be involved in the biosynthesis of terpenoids. The MOK_00957 encoding phytoene dehydrogenase was conserved only in a few *Pseudomonas* strains which mainly belonged to the species of *P. chlororaphis*. Its amino acid sequence showed 89% identities with coverage of 99% to that found in an uncultured proteobacterium QS1, and showed 37% identities with more than 94% coverage to that found in *Streptomyces*. But their capacity of synthesizing terpenoids has not been experimental verified. And the last non-ribosomal peptide synthase gene (*pvdL*) together with some strain-specific genes existed in MD9 also deleted. The *pea* genes encoding quinol hemoprotein amine dehydrogenase and related functional proteins in response to primary amines to provide the host with carbon and energy source [46], were removed together with the nearby genes in MD12. The operon *rmd-gmd-wbpW-wzm-wzt-wbpX-wbpY-wbpZ* in MD13 can be found in both pathogen and nonpathogenic strains, which has been proved to be responsible for the assembly of rhamnan polysaccharide according to the phenotype of *P. aeruginosa* mutants (*wbpX*, *wbpY* and *wbpZ*) [47]. And genes related to amino acid transport, carbohydrate metabolism, inorganic ion transport, or signal transduction system were removed in MD10, MD11 and MD14–18.

Finally, strain-specific genes without functional prediction and those encoding hypothetical proteins were deleted, including MD19–22. In MD21, there was a *czc* operon encoding a cation-proton antiporter, which is responsible for cobalt, zinc, and cadmium resistances [48].

### Characterization of deletion strains

**Fig. 2:**
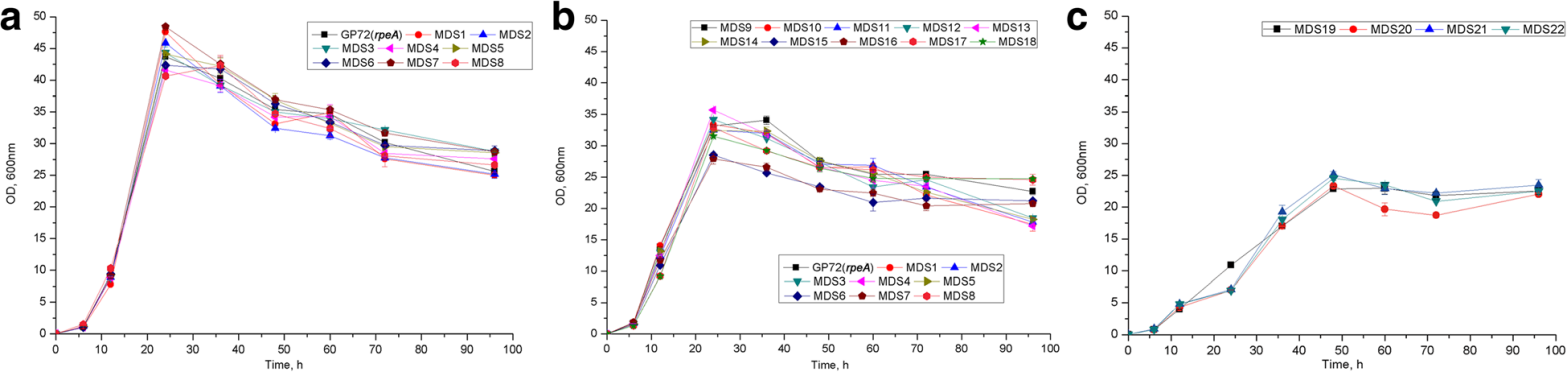
Growth curves of parent strain and genome-reduced mutants. Growth trends of all 23 strains were divided into three groups. **a** Group 1: GP72(*rpeA*-) and MDS1–8. **b** Group 2: MDS9–18. **c** Group 3: MDS19–22. Strains were grown in KMB liquid medium adding 40 mg/L Km at 28 °C and 180 rpm. The values are presented as the means of three replicates, and the error bars indicate the standard deviation (SD)

Growth features of MDS strains and the parent strain GP72(*rpeA*-) were first compared in fermentation condition. The growth curves can be divided into three groups based on features of the maximum cell population: Group 1 (including GP72(*rpeA*-) and MDS1–8) (Fig. 2a), Group 2 (including MDS9–18) (Fig. 2b) and Group 3 (including MDS19–22) (Fig. 2c). Strain MDS7 belonging to Group 1 with a 153.6 kb reduced genome exhibited robust cell growth. Strains in Group 2 had a slightly reduced growth, while mutants in Group 3, such as MDS19 showed a dramatically slower growth patterns than that of the parent strain.

**Fig. 3:**
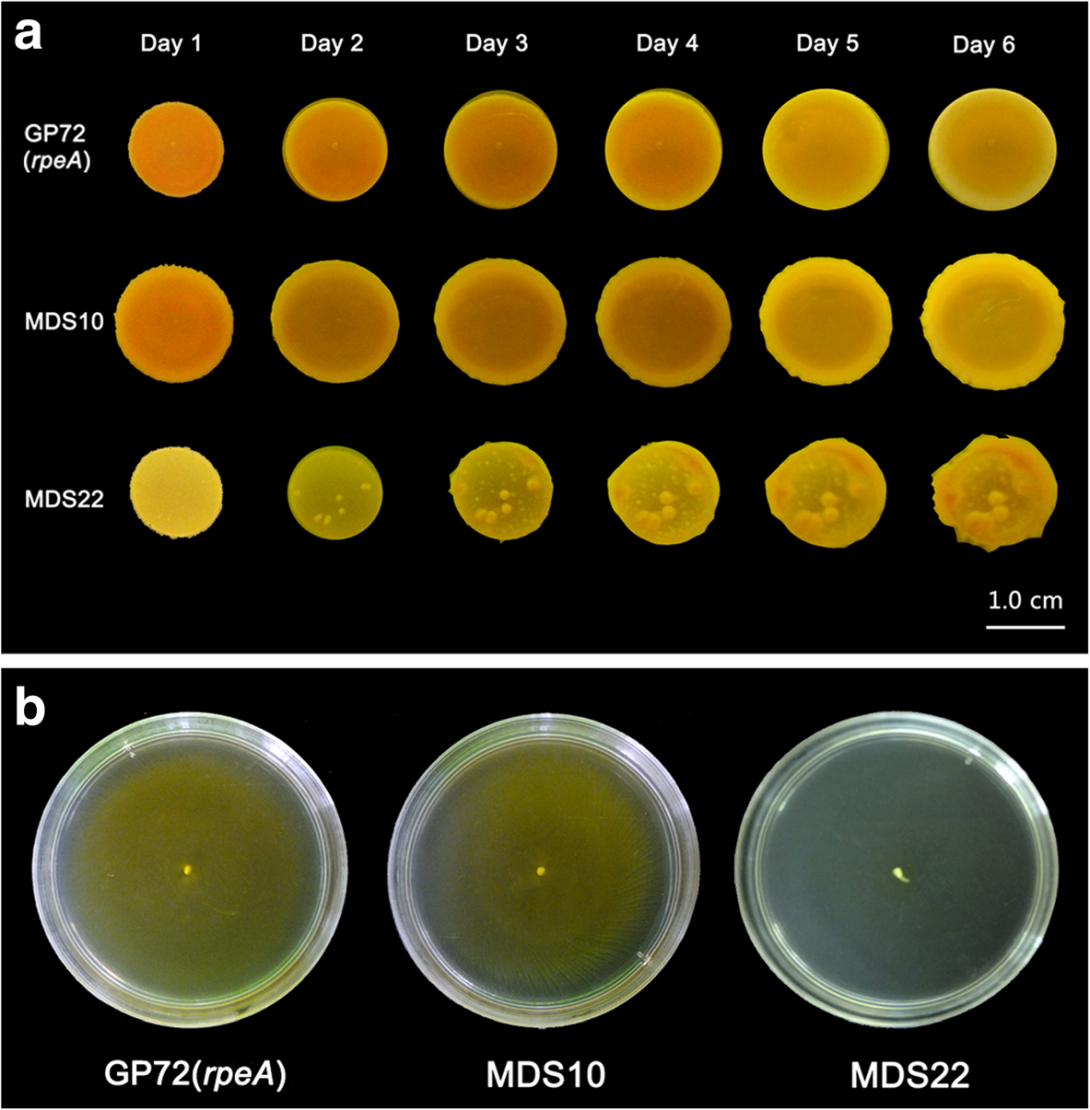
Phenotypic characterization of the parent strain GP72(*rpeA*-) and the genome-reduced mutants (MDS10 and MDS22). **a** Colony morphology assays were performed on 10 g/L tryptone broth plates (1% agar) containing Congo Red (40 μg/ml) and Coomassie Brilliant Blue (20 μg/ml). Strains were grown in LB liquid medium to late exponential phase, and then 10 μl were spotted onto plates and incubated at 28 °C for 6 days [49]. **b** Flagellar swimming assays were performed by using semi solid agarose plates. The swimming status was photographed after overnight incubation at 28 °C [51]. Three replicates were used for each sample and experiments repeated for twice

Here we observed colony morphologies of GP72(*rpeA*-) and its derivate mutants. Dietrich et al. [49] reported that the colony morphology could be modulated by phenazine production in *P. aeruginosa* strains, for the overproducer remained smooth after 6 days, while the phenazine null strain started to wrinkle earlier than the wild type. Phenotypes of representative strains with different levels of phenazine production are portrayed in Fig. 3a. The colonies of GP72(*rpeA*-), MDS10 and MDS15 remained smooth, but low-producing strain MDS22 started to wrinkle on the second day and severely wrinkled in the 6th day. The rugose colonial morphology also occurred in MDS19–21.

Similarly, motility is one of the physiological characteristics of microorganism and plays important roles in pseudomonads, such as access to nutrient effectively, avoidance of toxic substances, and dispersal in the optimal environment [50, 51]. Mutants MDS1–18 and strain GP72(*rpeA*-) moved rapidly across the agar surface and covered the entire surface after 24 h, and there was no significant difference among them, whereas strains MDS19–22 showed diminished or absent swimming abilities.

### Ability of producing secondary metabolites in GP72(*rpeA-*) and its derivate mutants

**Fig. 4:**
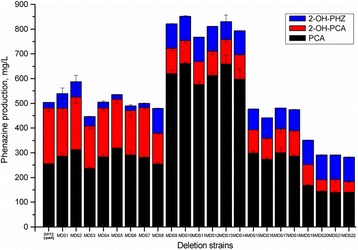
Production of phenazines by the parent strain and genome-reduced mutants. Production of PCA, 2-OH-PCA, and 2-OH-PHZ by the parent strain GP72(*rpeA*-) and its mutants after 24 h of fermentation in KMB medium at 28 °C and 180 rpm. The phenazines were extracted and measured by HPLC at a wavelength of 254 nm. The bar indicates the group mean and the error bar indicates SD from triplicate experiments

Three phenazine derivatives: PCA, 2-OH-PCA, and 2-OH-PHZ were analyzed in *P. chlororaphis* strains and results are displayed in Fig. 4. The 2-OH-PHZ is derived from 2-OH-PCA without the involvement of any enzyme [52], and PCA is converted to 2-OH-PCA via an aromatic monooxygenase encoded by *phzO* [33]. After 24 h of cultivation, the production of 2-OH-PHZ of MDS10 and MDS22 were 98.3 and 99.1 mg/L, respectively, both of which were 4.4 times higher than that of GP72(*rpeA*-) (22.2 mg/L) (*p* < 0.01). The significantly improved production was first occurred in MDS8, which was 5.6 times higher than that of the parent strain (*p* < 0.01). Notably, MDS10 had the highest production of PCA and total phenazines, 660.4 mg/L and 852.0 mg/L, respectively. Unfortunately, the PCA production showed varying degrees of decline in certain mutants, like MDS15 and MDS19.

Table 2 summarized the fermentation characteristics of the three representative strains GP72(*rpeA-*), MDS10 and MDS22 during the fermentation stage of 14 to 24 h. Compared with the parental strain, the specific growth rates of the mutants decreased, while the specific PCA production rates increased 3.3-fold (*p* < 0.01) and 1.2-fold (*p* < 0.05) in MDS10 and MDS22, respectively, and the specific total phenazine production rates changed similarly. Notably, the specific production rates of intermediate 2-OH-PCA in MDS10 and MDS22 were lower than that in the parental strain GP72(*rpeA-*) (p < 0.01), but the specific production rates of the final product 2-OH-PHZ significantly increased in mutants (p < 0.01), especially in MDS22 (11.5-fold).

**Table 2:** Fermentation characteristics of the parental strain GP72(*rpeA-*) and the genome-reduced mutants MDS10 and MDS22

Strain	Specific growth rate (h^−1^)	Specific PCA production rate(mmol/g/h)	Specific 2-OH-PCA production rate(mmol/g/h)	Specific 2-OH-PHZ-24 production rate(mmol/g/h)	Specific total phenazine production rate (g/g/h)^a^
GP72(*rpeA-*)	0.1226 ± 0.0034	0.0061 ± .00031	0.0064 ± 0.00024	0.0008 ± 0.00002	0.0031 ± 0.00011
MDS10	0.0601 ± .00360	0.0202 ± .00067	0.0015 ± 0.00010	0.0035 ± 0.00014	0.0056 ± 0.00019
MDS22	0.0314 ± .00280	0.0076 ± .00075	0.0025 ± 0.00015	0.0092 ± 0.00040	0.0039 ± 0.00025

Data represent the mean ± SD from three independent cultures

^a^Because the molecular weights of PCA, 2-OH-PCA and 2-OH-PHZ are different, the specific total phenazine production rate was calculated in grams

### Biolog phenotype microarray analysis of selected deletion strains

**Fig. 5:**
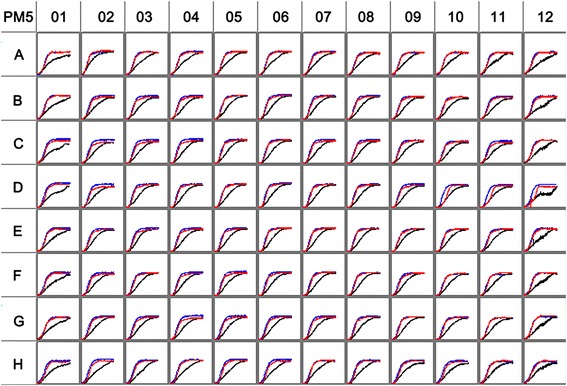
Metabolism of strains GP72(*rpeA*-), MDS10 and MDS22 on Biolog PM5 plates**.** Suspensions were added to each well of the PM5 microplates at a volume of 150 μl and incubated at 28 °C for 72 h [86]. Red and blue curves denote growth trends of GP72(*rpeA*-) and MDS10, respectively, while the black one shows information of MDS22, which indicates a significant defect in substance utilization. Wells A1 and A2 are negative control and positive control, respectively. The information of other 94 wells is listed in Additional file 6

The three strains GP72(*rpeA*-), MDS10 and MDS22 were analyzed for differences in biosynthetic pathways using Biolog phenotype microarrays [53]. Biolog PM5 plate containing different nutrient supplements was used, and the kinetic curve displayed the growth phenotype (Fig. 5). Strains MDS10 and GP72(*rpeA*-) had highly comparable kinetic curves, while strain MDS22 showed decreased substrate utilization capability. MDS10 and the parent strain showed identical growth characteristics in the negative well (minimal medium F-0 GN base without adding any supplement) or in the F8 well (IF-0 GN base with glucose). When supplied with substrates, such as L-serine, L-threonine, L-tryptophan, L-citrulline, cytosine, cytidine, 2′-deoxy cytidine and *p*-amino-benzoic acid, the growth of MDS10 has improved as compared to the parental strain GP72(*rpeA-*).

## Discussion

Previous studied showed that numbers of essential genes vary among different genera, species, strains, even same strain in different studies. This difference might result from mutant complementation, different cultural conditions [54] and different study strategies, like different transposons [4]. Therefore, we chose the overlapping set of conserved genes as candidate essentials in the genome of GP72, which showed a certain degree of consistency with the minimal bacterial gene set comprising 206 protein-coding genes proposed by Gil et al. [55]. The biggest difference is the transport genes, for they only have a PTS for glucose and *pitA* to provide the phosphate. Because the authors recognized it is hard to define specific transporters depending on the nutrients available in the environment or the cell membrane characteristics [55]. The data set of essential genes in this study provides frameworks for further research, such as optimizing the metabolic network or constructing chassis cells. And the exactly essential genes in GP72(*rpeA*-) under certain conditions should be verified by targeted gene deletions [2], saturation transposon mutagenesis [5] or RNA interference [56].

Here we used homologous recombination strategy to implement large-scale genome reduction to develop series sequential deletion mutants. The method we used was suitable to construct large-scale deletions. The largest deleted region covered more than 87 kb. The 5.977-Mb genome of MDS22 is smaller than any genome of *P. chlororaphis* strains in the available genomic data (6.66 to 7.30 Mb) [23]. Other large fragment deletion strategies were also widely used in *Pseudomonas*, such as Flp/FRT, Cre/*loxP* and I-SceI site-specific recombination systems [57–59]. While Flp/FRT and Cre/*loxP* systems cannot generate the true scarless mutant, leaving one copy of *frt* or *loxP* site in the genome, and the foreign sequences may interfere with gene expression or subsequent deletions.

In this study, we aimed to construct *Pseudomonas* mutants with less energy consumption and higher productivity from the perspective of synthetic biology and its applications. So genes that were unessential when bacteria in nutrient-rich medium were selected for the deletion, The previous study showed that the achromobactin operon is expressed under iron deficient conditions [41] and pyoverdine biosynthesis genes are unessential in *P. aeruginosa* [4]. Although pyoverdine is a high-affinity siderophore under the iron starvation condition, which is beneficial for the growth of strain; but on the other hand, its biosynthesis and excretion also require much energy and amino acids [60]. And *Pseudomonas* could also get iron via other mechanisms, such as the strain *P. stutzeri* A1501 [36].

Deletions of multiple futile metabolic pathways may be useful to save cellular energy and resources and thereby improve the growth of MDS7. The reduced growth rate might be due to the deletion of genomic loci MOK_00502, MOK_00503 (encoding PvdL and PvdS, respectively), MOK_00505 (encoding amino acid ABC transporter substrate-binding protein) and MOK_00506–00507 (encoding a repressor AtuR and a protein AtuA related to acyclic terpene utilization) in MD9, and MOK_03695 (related to transcriptional regulator) and MOK_03714–03716 (encoding outer membrane receptor protein, Fe^2+^-dicitrate sensor and RNA polymerase sigma factor, respectively) in MD19. PvdL is the core enzyme and necessary for the production of pyoverdine [61]. The deletion of MD3 may not affect the synthesis of siderophore, for the strain can use isoenzymes on the genome. But when MD9 containing *pvdL* was deleted*,* MDS9 may have defect in iron acquisition, resulting in slower growth trend than that of MDS3. As a *pvdL* mutant derived from *P. fluorescens,* SBW25 displays reduced cell density when compared to the wild-type in iron depleted medium [62]. Besides, the cumulative effect of consecutive deletions may play an important role in the growth of MDS19. *E. coli* ∆16 lacked up to 29.7% of the parental chromosome grew more slowly than the parent strain MG1655 [13], whereas the mutant reduced the genome size by 7% exhibited normal growth [63].

Besides the reduction of phenazine production, the rugose colonies may be due to the overproduction of an exopolysaccharide [64] or an extracellular cellulose-like material [65], or the metabolism of carbohydrates [66]. The swimming character mainly depends on flagella [50], but the deletion in MD19 did not contain any biosynthetic genes of flagella. We speculate that the defects may occur at the functional level, such as defects in chemotaxis and the cytoplasmic signal transduction system [51, 67], which may be under the control of GntR family (MOK_03695) and TetR family (MOK_03712) transcriptional regulator. Overall, phenotypic differences between the two types of strains: rough colonies with defective flagellar motility and smooth colonies with normal flagellar activity were performed by the results found in *P. aeruginosa* 57RP [68]. The exact reasons for these different phenotypic traits need further research.

Comprehensive observation of the growth and phenazines production in representative strains: GP72(*rpeA*-), MDS10 and MDS22, we found mutants grew slower than the parent strain, but the production capacities of phenazines, including initial product and the final one, were improved greatly. So keeping the specific growth rate at a certain level may facilitate the productivity of secondary metabolites. The higher 2-OH-PHZ production firstly occurred in mutant MDS8, which is produced abiotically from 2-OH-PCA [52]. The deletion of MD8 contained genes encoding quorum sensing system QsyI/R (MOK_00967 and MOK_00968) which may affect the production of phenazines. QsyR is a LuxR family transcriptional regulator, and its amino acid sequence was conserved among *P. chlororaphis*. The sequence in strain GP72(*rpeA*-) showed 54% identity to that found in *P syringae*. In *P syringae*, it coordinates regulation of pathogenesis-related genes, but the mechanism has not been illustrated [69]. It also showed 31% identity to the amino acid sequence of *qscR* found in *P. aeruginosa*. QscR is a negative regulator that could repress the transcription of phenazines [70]. The increased total phenazines production may be from the deletion of *pvdL* in MD9. The *pvdL* gene encodes a non-ribosomal peptide synthetase involved in incorporating L-glutamate, D-tyrosine and L-2,4-diaminobutanoate in a precursor peptide chain [71]. The elimination of this gene may save cellular resources and energy, and facilitate the synthesis of phenazines.

The MDS15 as the progenitor of following low-producing strains lacked genes of *apr* gene cluster encoding alkaline protease (AprA), its inhibitor (AprI) and secretion system (AprDEF). These genes showed high identities to those found in other pseudomonads. Previous studies showed that endoproteases including alkaline protease were involved in the regulation of numerous cellular processes by degrading specific proteins [72], while AprI is a highly potent inhibitor to protect periplasmic proteins from the AprA proteolysis [73]. The protease secretion mechanisms among different species, such as *Pseudomonas* and *Erwinia*, are quite similar [74]. Another protease namely serine protease Lon has been proved to be a powerful negative regulator, and the mutant can greatly improve the pyoluteorin production [75]. So the relationship between *AprA* and phenazine production is unclear and needs further study. Besides, it also lacked genes of *dmpA* (MOK_02632), which catalyzes peptide to release the N-terminal D and L amino acids. However, in MDS19 and other strains, the reduced concentrations may result from the interruption of the transport system.

In PM assays, we found that the substrates could make the growth characteristics difference between mutant MDS10 and the parental strain. Previous studies showed that some of those substrates also influence the production of phenazines [76]. For example, phenazine-1-carboxamide production was improved by adding individual amino acids threonine and tryptophan in *P. chlororaphis* PCL1391 [76]. And *p*-amino-benzoic acid was used to suppress phenazine production during triparental matings in *P. aureofaciens* 30–84 [77]. Further work is needed to check the importance of these substrates in the performance of *Pseudomonas chlororaphis* mutants.

## Conclusions

The genome reduction strategy in this study is useful to construct *Pseudomonas* and other microbial cell factories for the production of valuable metabolites. *P. chlororaphis* strain GP72(*repA*-) showed the feasibility of constructing genome-reduced *Pseudomonas* strains. In the next step, the genome-scale metabolic reconstruction as well as regulatory network analysis will give more information to construct a desirable chassis cell with system simplicity and practicability for biotechnological application.

## Methods

### Bacterial strains, plasmid and culture conditions

For constructing genomic deletion strains, we used *P. chlororaphis* GP72(*rpeA*-) as the parent strain, which derives from inactivation of *rpeA* from strain GP72 and shows stable and high 2-OH-PHZ production [33]. *E. coli* DH5ɑ (TransGen Biotech, Beijing, China) was used to construct series deletion strains, and *E. coli* S17–1 (λpir) served as the donor strain to transform plasmids to *P. chlororaphis* in conjugations [78]. The plasmid pK18 *mobsacB* used to harbor homologous fragments with the target sequences deriving from pK18 is a broad-host-range vector containing genetic loci *sacB* and Km^r^ (accession No. FJ437239) [79]. Bacterial strains and the plasmids used in this study are summarized in Table 3. All studied *Pseudomonas* strains were grown in Luria-Bertani (LB) medium (tryptone 10.0 g, yeast extract 5.0 g, NaCl 10.0 g/L) or King’s medium B (KMB) (glycerol 20 g, tryptone 20 g, MgSO4 0.732 g, K_2_HPO_4_ 0.514 g/L) at 28 °C and 180 rpm, while *E. coli* strains were cultured in LB medium at 37 °C and 220 rpm. If required, antibiotics in the medium were used at the following concentrations: 40 mg/L kanamycin (Km), 100 mg/L gentamicin (Gm), 24 mg/L isopropyl-*β*-D-1-thiogalactopyranoside (IPTG), 40 mg/L 5-bromo-4-chloro-3-indolyl-beta-D-galactopyranoside (X-gal), and 15% (*w*/*v*) sucrose.

**Table 3:** Main bacterial strains and plasmids used in this study

Bacterial strain or plasmid	Relevant characteristics^a^	Source or reference
*Escherichia coli*		
DH5α	Cloning host; F^−^ λ^−^ *endA1 glnX44*(AS) *hiE1 recA1 relA1 spoT1 gyrA96*(Nal^R^) *rfbC1 deoR nupG* Φ80(*lacZ*Δ*M15*) Δ(*argF*-*lac*)*U169 hsdR17*(*r*_*K*_^−^ *m*_*K*_^*+*^)	TransGen Biotech, Beijing, China
S17–1 (λpir)	Donor strain; *res* ^*−*^ *pro mod* ^*+*^ integrated copy of RP4, mob^+^, used for incorporating constructs into *P. chlororaphis*	Lab stock; Hoffmann et al.
*Pseudomonas chlororaphis*		
GP72	*P. chlororaphis* GP72 wild-type strain	Lab stock; Liu et al.
GP72(*rpeA*-)	Gm^R^, *rpeA* insertionally inactivation mutant of GP72	Lab stock; Huang et al.
MDS5	Gm^R^, mutant of GP72(*rpeA*-) with deletion of 1.40% of the genome, including five secondary metabolic gene clusters	This study
MDS10	Gm^R^, mutant of GP72(*rpeA*-) with deletion of 3.52% of the genome	This study
MDS22	Gm^R^, mutant of GP72(*rpeA*-) with deletion of 10.29% of the genome	This study
Plasmid		
pK18 *mobsacB*	Broad-host-range gene replacement vector, *sacB*, Km^R^	Lab stock; Schafer et al.
pK18- MDX^b^	pK18 *mobsacB* containing MDX flanking region	This study

^a^Antibiotic markers: Km, kanamycin

^b^MDX means the corresponding multiple-deletion region

### Selection of deletion targets

For comparative genomic analysis, we used the genome sequence of *P. chlororaphis* GP72 (accession No. NZ_AHAY01000000) [80]. All predicted protein sequences and genome annotations were obtained from the integrated microbial genomes system [81]. BLASTP programs were conducted between protein sequences of strain GP72 and *P. stutzeri* A1501 (accession No. CP000304) [36] using the mGenomeSubtractor (http://202.120.12.134/mGS2/) [38]. The strain-specific genes were identified under a homology value cutoff of 0.42 at E-value < 10^−5^ [38]. The regions with continuous strain-specific genes were selected as elimination candidates.

For assessing these selected genes, we identified essential genes existing in strain GP72. There are 33 prokaryotic organisms in total listed on the database of essential genes (DEG) version 10.5 [6], including 23 g-negative bacteria. Since there are multiple bacterial records for one species and significant overlap among sets of essential genes derived from gram-negative bacteria [82, 83], we chose the newest data set for each species in this study. For example, the recent set of *P. aeruginosa* essential genes is more accurate based on combining gene information from both PAO1 and PA14 insertion libraries [4]. Besides, previous studies show that predicting essential genes via homology searches are significantly affected by phylogenetically unrelated organisms [84]. Therefore, 15 sets of essential genes were selected among gram-negative bacteria and used to create local databases. The local BLASTP programs were carried out on NCBI BLASTP+ version 2.2.30+. The homology searches were launched between protein coding sequences of GP72 and each local database to identify essential genes in *P. chlororaphis*. These were carried out under an E-value cut-off of 10^−10^ and 35% identity threshold [40]. Finally, five known secondary metabolite clusters, and 17 strain-specific regions larger than 15 kb and containing more than 10 unnecessary continuous genes were selected for deletion [14].

### Construction of multiple deletion strains

For DNA manipulations, plasmid DNA and genomic DNA were isolated from *E. coli* using a spin plasmid preparation kit and *P. chlororaphis* using a genomic DNA isolation kit, respectively, according to the manufacturer’s instructions (TransGen Biotech, Beijing, China). All the enzymes were purchased from TaKaRa (Dalian, China) except for Taq polymerase (TransGen Biotech, Beijing, China). All primers used in this study were synthesized at Shanghai Sunny Biotechnology Co, Ltd. (Shanghai, China) and are listed in Additional file 4. DNA products were gel purified using AxyPrep DNA gel extraction kit (Corning, Shanghai, China). DNA sequencing was conducted at BGI Tech Solutions Co., Ltd. (Shenzhen, China) and sequences were analyzed on the NCBI website.

Gene deletions in *P. chlororaphis* strain were conducted using a markerless deletion method with pK18*mobsacB* as described previously [85]. The method was slightly modified based on the specific strain (Additional file 5). Firstly, the upstream and downstream homologous arms ranging from 400 to 800 bp were amplified from the parent strain genomic DNA using two pairs of primers (F1 and R1) and (F2 and R2), respectively. Two primers of R1 and F2 had an 18–20 bp homology region between them. So purified polymerase chain reaction (PCR) fragments were joined by an overlap PCR using primers of F1 and R2, both of which contained different restriction sites at their 5′ ends. The purified DNA product was then digested and cloned into the plasmid pK18 *mobsacB*. Secondly, the ligation and purified controls were transformed into chemically competent DH5ɑ cells using standard protocol followed by blue/white screening on LB plates containing Km X-gal and IPTG. The selected white colonies (Km^R^) were verified by PCR amplification and DNA sequencing. Thirdly, the deletion construct was transformed into *E. coli* S17–1 (λpir), resulting in the S17–1 (λpir) harboring the construct (Km^R^Gm^S^) and then conjugated it into *P. chlororaphis* to get single crossover merodiploid transconjugants, which were selected on LB plates with antibiotics Km and Gm. At each step, the positive clones were confirmed by PCR amplification using primers of F1 and R2. Finally, merodiploid (Km^R^Gm^R^) were transferred to LB agar plates with 15% (*w*/*v*) sucrose to counter-select the integration, which resulted in the loss of the *sacB* gene. Suc^R^Gm^R^Km^S^ colonies were selected and screened by PCR using primers specific to sequences in the remaining genome (F1 and R2) and deleted fragment (NF and NR). Then the positive clones were also confirmed by spotting them on LB plates only containing Km, on which clones cannot grow. The subsequent deletion mutants were constructed based on the same strategy as mentioned above.

### Fermentation processing

A single colony was selected and grown in a test tube containing 5 mL KMB liquid medium supplemented with 40 mg/L Km at 28 °C overnight. Then the seed culture was transferred to 50 mL KMB medium in a 250 mL baffled flask at an OD_600_ of 0.02. The fermentation process was carried at 28 °C and 180 rpm during 4 days. Cultures were collected for the measurement of OD_600_, biomass, and production of phenazine compounds. There are triplicate experiments for each fermentation test, and experiments were repeated twice. The result value is expressed as the mean ± SD. Statistical significances of the differences between groups were examined by Student’s t test, using paired data.

### Quantification of phenazine compounds

Samples were prepared following the previous procedure [33]. The fermentation broth was adjusted to pH 2.0 using 6 M HCl and extracted with three volumes of ethyl acetate by vigorous shaking. The organic layer was then mixed with 1/10 volume of distilled water and shaken vigorously. Finally, the organic phase containing phenazine compounds was evaporated under vacuum pressure. The phenazine compounds were dissolved in methanol for further analysis. The analysis was carried out by using the Agilent 1260 Infinity high-performance liquid chromatography (HPLC) series with a C-18 reverse phase column and ultraviolet light detector (Agilent Technologies, USA). The mobile phase changed during the detection process methanol: ammonium acetate =20:80 (*v*/v) in the first 5 min, then changing to 50:50 in 5 to 25 min and 20:80 again in the last 5 min (25–30 min). The retention times for PCA, 2-OH-PCA, and 2-OH-PHZ were approximately 9.1 min, 13.3 min and 21.7 min, respectively.

### Morphology assays

Colony morphology assays were performed as previously described by using 10 g/L tryptone broth plates (1% agar) with the addition of Congo Red (40 μg/ml) and Coomassie Brilliant Blue (20 μg/ml) [49]. Strains were grown in LB medium to late exponential phase, and then 10 μl were spotted onto plates and incubated for 6 days. Flagellar swimming assays were done by stabbing tryptone swim plates (1% tryptone, 0.5% NaCl, 0.3% agar) as described previously [51]. These plates were inoculated with bacteria from an overnight culture on LB agar plates using a sterile toothpick. The swimming status was photographed after overnight. All plates were wrapped with saran to prevent dehydration and cultured at 28 °C.

### Biolog phenotype microarray

The biosynthetic pathways of strain *P. chlororaphis* GP72(*rpeA*-), MDS10 and MDS22 were compared using phenotype microarray (PM) technology in plate PM5 containing 94 different nutrients (BIOLOG Inc. CA, USA). All procedures were performed as described by Bochner [86]. The tested strains were grown overnight at 28 °C using Biolog Universal Growth agar (BIOLOG Inc. CA, USA) plates. The single colony was swabbed from the plate and suspended using IF-0 GN base inoculating fluid (BIOLOG Inc. CA, USA) to a density of 85% transmittance in the Biolog turbidimeter. Suspensions were added to each well of the PM5 microplates at a volume of 150 μl and incubated at 28 °C for 72 h. Plates were placed in the OmniLog instrument, and cell growth was recorded by the respiration-dependent color change of tetrazolium violet every 15 min. Finally, the readouts were analyzed based on the different kinetic curves reflecting the phenotypic changes. The added nutrient for each well is listed in Additional file 6. The PM assay was performed twice.

## Additional files

Additional file 1: Predicted essential genes of *P. chlororaphis* strain. (XLSX 29 kb)

Additional file 2: Homology search of *P. chlororaphis* essential genes. Essential genes in the genome of *P. chlororaphis* were predicted using homology searches based on local BLASTP program. (TIFF 169 kb)

Additional file 3: Deleted genes in each MD region. (XLSX 51 kb)

Additional file 4: Primers used in this study. (XLSX 14 kb)

Additional file 5: Flowchart of the markerless deletion method based on pK18*mobsacB*. (TIFF 110 kb)

Additional file 6: Nutrient added in each well of the Biolog PM5 plate. (XLSX 12 kb)

## Abbreviations

2-OH-PCA: 2-hydroxyphenazine-1-carboxylic acid
2-OH-PHZ: 2-hydroxyphenazine
BLAST: Basic local alignment search tool
bp: Base pairs
COG: Cluster of orthologous group
DEG: Database of essential genes
Gm: Gentamicin
HPLC: High-performance liquid chromatography
IPTG: Isopropyl-β-D-1-thiogalactopyranoside
Km: Kanamycin
KMB: King’s medium B
LB: Luria-Bertani
MD: Multiple-deletion
MDS: Multiple-deletion series
PCA: Phenazine-1-carboxylic acid
PCR: Polymerase chain reaction
PM: Phenotype microarray
Pvd: Pyoverdine
SD: Standard deviation
X-gal: 5-bromo-4-chloro-3-indolyl-beta-D-galactopyranoside
