# Supplementary figures and images for: Developing genome-reduced Pseudomonas chlororaphis strains for the production of secondary metabolites
Source: BMC Genomics. 2017 Sep 11;18:715. doi: 10.1186/s12864-017-4127-2 (PMC5594592; doi:10.1186/s12864-017-4127-2)

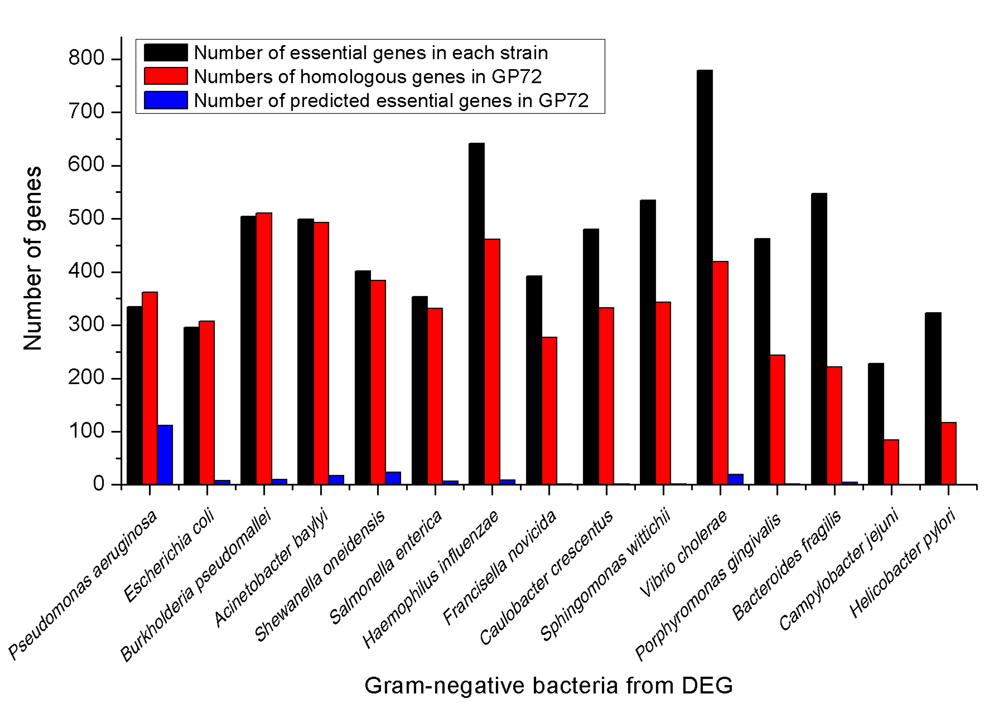

Supplement: Supplementary file 2 — Homology search of P. chlororaphis essential genes. Essential genes in the genome of P. chlororaphis were predicted using homology searches based on local BLASTP program. (TIFF 169 kb) [file 12864_2017_4127_MOESM2_ESM.tif]

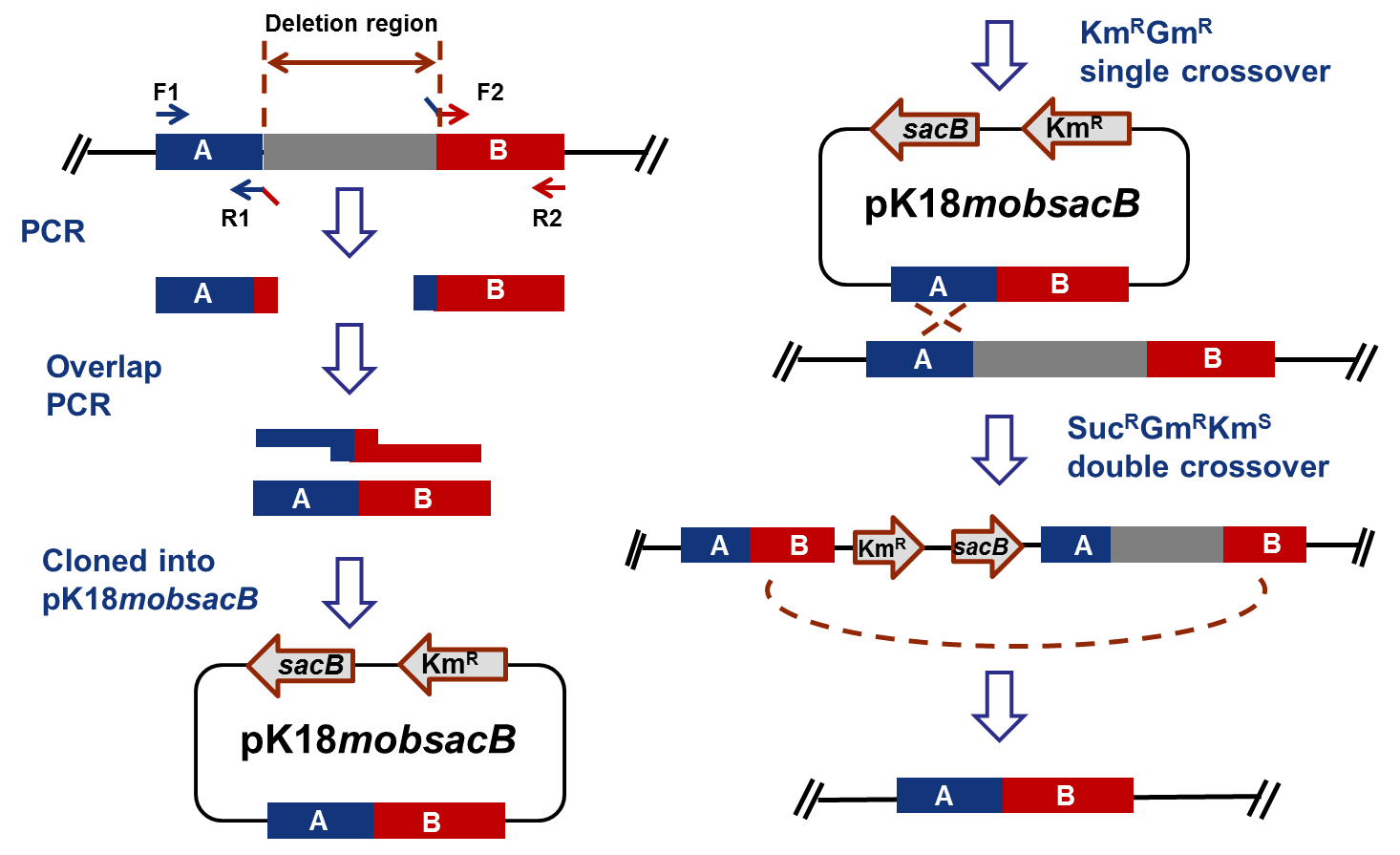

Supplement: Supplementary file 5 — Flowchart of the markerless deletion method based on pK18mobsacB. (TIFF 110 kb) [file 12864_2017_4127_MOESM5_ESM.tif]
